# Lipid-Based Nanoparticles in Cancer Diagnosis and Therapy

**DOI:** 10.1155/2013/165981

**Published:** 2013-07-09

**Authors:** Andrew D. Miller

**Affiliations:** ^1^Institute of Pharmaceutical Science, King's College London, Franklin-Wilkins Building, Waterloo Campus, 150 Stamford Street, London SE1 9NH, UK; ^2^GlobalAcorn Ltd., London, UK

## Abstract

Today, researchers are constantly developing new nanomaterials, nanodevices, and nanoparticles to meet unmet needs in the delivery of therapeutic agents and imaging agents for cancer therapy and diagnosis, respectively. Of particular interest here are lipid-based nanoparticles (LNPs) that are genuine particles (approximately 100 nm in dimension) assembled from varieties of lipid and other chemical components that act collectively to overcome biological barriers (biobarriers), in order for LNPs to preferentially accumulate in or around disease-target cells for the functional delivery of therapeutic agents for treatment or of imaging agents for diagnosis. The capabilities of these LNPs will clearly vary depending on functional requirements, but the nanoscale allows for an impressive level of diversity in capabilities to enable corresponding LNPs to address an equally diverse range of functional requirements. Accordingly, LNPs should be considered appropriate vehicles to provide an integrated, personalized approach to cancer diagnosis and therapy in future cancer disease management.

## 1. Introduction

Unmet medical needs in cancer diagnosis and therapy remain substantial in spite of decades of research. On the other hand, there are substantial numbers of potentially potent therapeutic agents available (both biopharmaceutical and small molecule drug related) that are either too large in size, too highly charged, too metabolically unstable, and/or too insoluble to reach cancer target cells without the assistance of delivery “vehicles.” Nowadays, this situation is seen to be an opportunity for cancer nanotechnology, a field that seeks to take a multidisciplinary, problem-driven approach to research that cuts across the traditional boundaries of biology, chemistry, engineering, and medicine with the aim of using nanotechnology to bring about major advances in cancer detection, diagnosis, and treatment [1–4]. In particular cancer nanotechnology could leverage an opening up of 1000s of new potential disease targets for therapeutic intervention by enabling the functional delivery of new classes of therapeutic agents to target cells. Following this there is the eventual likelihood that cancer nanotechnology could also open up opportunities for personalised cancer diagnosis and treatment regimes [3], by means of multifunctional nanoparticles for (a) the detection of cancer disease-specific biomarkers, (b) the imaging of tumours and their metastases, (c) the functional delivery of therapeutic agents to target cells, and (d) the real-time monitoring of treatment in progression. If this is the potential, how close are we really? 

**Figure 1:**
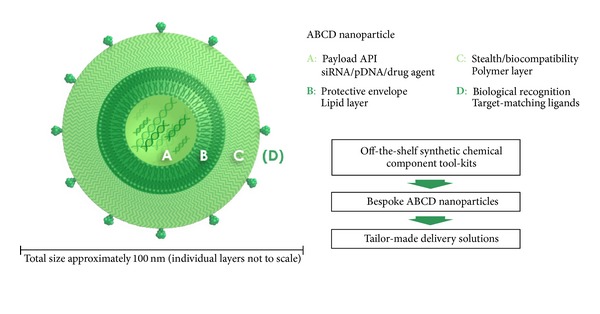
Active pharmaceutical ingredient (API; therapeutic bioactive or intractable drug) condensed within functional concentric layers of chemical components making up nanoparticle structure designed to enable efficient delivery (trafficking) of active therapeutic agent to target cells (used with permission of GlobalAcorn Limited).

Where nanoparticles are to be created for the functional delivery of imaging and/or therapeutic agents, many factors have to be taken into consideration. This fact can be illustrated with reference to the fields of gene therapy and RNA interference (RNAi) therapeutics where lipid-based nanoparticles (LNPs) have been devised for functional delivery of therapeutic nucleic acids with some success. When LNPs have been designed successfully and used to mediate the functional delivery of therapeutic nucleic acids *in vivo*, these LNPs conform typically to the ABCD nanoparticle paradigm (Figure 1). According to this general paradigm, functional delivery nanoparticles consist of active pharmaceutical ingredients (APIs) (A-component) surrounded initially by compaction/association agents (B-components—lipids in this case) designed to help sequester, carry, and promote functional delivery of the A-component. Such AB-core nanoparticles may have some utility *in vivo* but more typically require coating with a stealth/biocompatibility polymer layer (C-component—most often polyethylene glycol (PEG)) designed to render resulting ABC nanoparticles with colloidal stability in biological fluids and immunoprotection from the reticuloendothelial system (RES) plus other immune system responses. Finally, an optional biological targeting layer (D-components—*bona fide* biological receptor-specific ligands) might be added to confer the resulting ABCD nanoparticle with target cell specificity. A key design principle here is that tailor-made LNPs can self-assemble reliably from tool-kits of purpose designed chemical components [5–15]. Accordingly, the concept of a personalized LNP formulation, assembled in the pharmacy for an individual patient does not seem so far removed from reality.

The ABCD nanoparticle paradigm represents a set of well-found principles of design that are being implemented in the real world with the formation of actual LNPs leading to actual demonstrated functional properties at least in pre-clinical studies. As such, the design principles laid out in the ABCD nanoparticle paradigm are widely corroborated in the literature [1, 16–24]. Clearly functional nanoparticles need to be constructed from a range of chemical components designed to promote functional delivery of different diagnostic and/or therapeutic agents *in vivo*. In practise this means that nanoparticles need to be equipped to overcome relevant “bio-barriers” in accordance with the pharmacological requirements of API use such as site, time, and duration of action. Importantly too, with clinical goals in mind, nanoparticles have to be considered differently to small and large molecular drugs. For instance, regulations from the FDA state that Absorption, Distribution, Metabolism and Excretion (ADME) studies need to be redesigned in the case of nanoparticles to take into consideration their aggregation and surface chemical characteristics [25]. 

In terms of cancer diagnosis and therapy, there is one factor that is very much in favour of multifunctional LNP use. LNPs administered in the blood stream (i.v. administration) frequently accumulate in tumours anyway due to the enhanced permeability and retention (EPR) effect, a behaviour that was identified by Matsumura and Maeda as a means to target anticancer therapeutic agents to tumours [26]. LNP accumulation in tumours takes place due to the presence of highly permeable blood vessels in tumours with large fenestrations (>100 nm in size), a result of rapid, defective angiogenesis. In addition tumours are characterised by dysfunctional lymphatic drainage that helps the retention of LNPs in tumour for long enough to enable local nanoparticle disintegration in the vicinity of tumour cells. The phenomenon has been used widely to explain the efficiency of nanoparticle and macromolecular drug accumulation in tumours [27]. Unfortunately, knowledge of LNP biokinetics, metabolism, and clearance is otherwise poor since too few LNP products have been clinically tested. This is a major limitation in the growth of the field of cancer nanotechnology. Nevertheless, cancer nanotechnology is a fast developing field and new data is arriving all the time. In the following sections, the status of LNP use in cancer diagnosis and therapy will be surveyed. 

## 2. Prototype Drug Nanoparticles for Cancer Therapy

The capacity of LNPs to be prepared by reliable, spontaneous self-assembly from purpose designed chemical components (most of which are lipids either natural or synthetic) is due to the unrivalled capacity of structural lipids in aqueous solution to undergo association and controlled assembly into potentially vast three-dimensional macromolecular assemblies. Selected structural lipids self-assemble into liposomes that are typically approximately 100 nm in diameter and consist of a lipid bilayer surrounding an aqueous cavity [28–30]. This cavity can be used to entrap water-soluble drugs in an enclosed volume resulting in a drug-AB nanoparticle [31, 32]. 

The first drug-AB nanoparticles reported were designed to improve the pharmacokinetics and biodistribution of the anthracycline drug doxorubicin. Doxorubicin is a potent anticancer agent but is cardiotoxic. In order to minimize cardiotoxicity, doxorubicin was initially encapsulated in anionic liposomes giving anionic doxorubicin-AB nanoparticles that enabled improved drug accumulation in tumours and increased antitumour activity while diminishing side effects of cardiotoxicity [33, 34]. Such drug formulations have been used efficiently in clinic for the treatment of ovarian and breast cancer [35, 36]. Thereafter, Doxil was devised corresponding to a drug-ABC nanoparticle system (PEGylated drug nanoparticle system), comprising PEGylated liposomes with encapsulated doxorubicin. These Doxil drug nanoparticles were designed to improve drug pharmacokinetics and reduce toxicity further by maximizing RES avoidance [37–39], making use of the PEG layer to reduce uptake by RES macrophages of the mononuclear phagocyte system (MPS) [40, 41].

In more recent times, prototype nucleic acid-AB, -ABC, or -ABCD nanoparticles have been tested for functional delivery of therapeutic nucleic acids to target cells in animal models of human disease (to liver for treatment of hepatitis B and C virus infection, to ovarian cancer lesions for cancer therapy) and to target cells in murine lungs [42–47]. Rules for enhancing efficient delivery through receptor-mediated uptake of nucleic acid-ABCD nanoparticles into target cells are also being studied and appreciated [48–50] (Wang, M. et al., J. Drug Del., 2013, paper in submission).

## 3. Prototype Imaging Nanoparticles for Cancer Imaging 

From the point of view of using LNPs for the imaging of cancer, the ability to combine imaging agents appropriately is central. In terms of the ABCD nanoparticle paradigm, the A-component now becomes an imaging agent(s) instead of a therapeutic agent. Potentially important preclinical studies have been carried out recently with imaging LNPs set up for positive contrast magnetic resonance imaging (MRI) [51, 52]. The first described LNPs of this class were formulated by trapping water-soluble, paramagnetic, positive contrast imaging agents (such as MnCl_2_, gadolinium (III) diethylenetriamine pentaacetic acid (Gd.DTPA), and the manganese (II) equivalent (Mn.DTPA)) in the enclosed volume of a liposome resulting in prototype lipid-based, positive contrast imaging LNPs [53, 54]. Disadvantages were quickly reported such as poor encapsulation efficiency, poor stability, and clear toxicities due to importune contrast agent leakage and poor relaxivity [55]. These problems were obviated when hydrophobic lipidic chains were “grafted” on to contrast agents, thereby enabling these agents to be hosted by a lipid bilayer [56]. Such lipidic contrast agents formulated in association with the bilayer of a liposome exhibit improved ionic relaxivity and therefore could be used for dynamic MRI experiments in mice *in vivo* [57]. 

**Figure 2:**
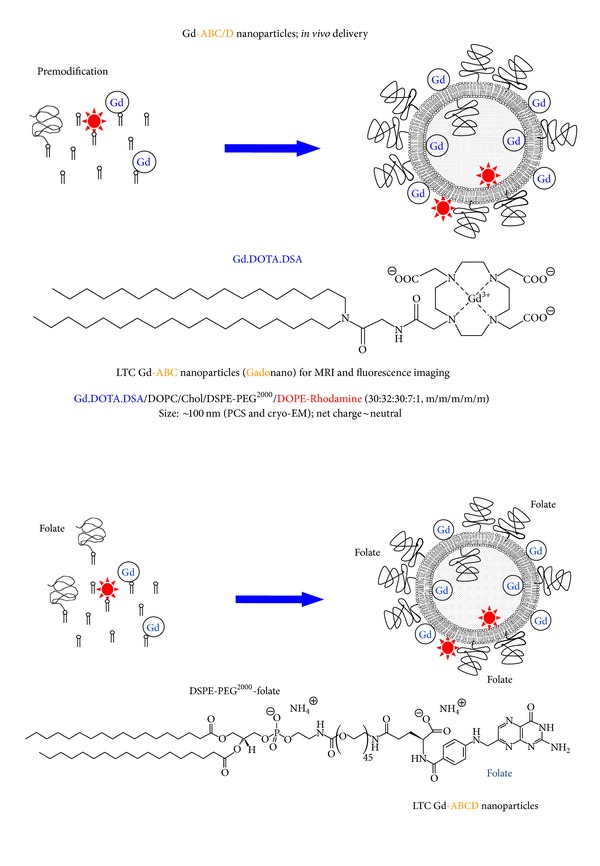
Schematic diagrams showing self-assembly of passively targeted Gd-ABC (top) and folate-receptor targeted Gd-ABCD nanoparticles (bottom) for IGROV-1 tumour imaging from combinations of structural lipids, PEG-lipids and imaging lipids [58, 59]. LTC: long-term circulation enabled by virtue of the use of bilayer stabilizing lipids and 7 mol% PEG-lipid in the outer leaflet membranes of nanoparticle structures.

A potentially significant variation on this theme involves gadolinium (III) ions complexed with 1,4,7,10-tetraazacyclododecane-1,4,7,10-tetraacetic acid (DOTA) to which hydrophobic lipidic chains are attached. In particular, gadolinium (III) 2-(4,7-bis-carboxymethyl-10-[(*N*,*N*-distearylamidomethyl-*N*′-amidomethyl]-1,4,7,10-tetraazacyclododec-1-yl)-acetic acid (Gd.DOTA.DSA) was prepared and formulated into passively targeted Gd-ABC (no biological targeting layer) and folate-receptor targeted Gd-ABCD nanoparticles in conjunction with a number of other naturally available and synthetic lipid components such as (*ω*-methoxy-polyethylene glycol 2000)-*N*-carboxy-distearoyl-L-*α*-phosphatidylethanolamine (DSPE-PEG^2000^) or its folate variant (DSPE-PEG^2000^-folate), and fluorescent lipid dioleoyl-L-*α*-phosphatidylethanolamine-*N*-(lissamine rhodamine B sulphonyl) (DOPE-Rhodamine) (Figure 2). These bimodal imaging LNP systems demonstrated excellent tumour tissue penetration and tumour MRI contrast imaging in both instances [58–60]. Interestingly, the folate-receptor targeted Gd-ABCD nanoparticles exhibited a 4-fold decrease in tumor *T*_1_ value in just 2 h after-injection, a level of tissue relaxation change that was observed only 24 h after administration of passively targeted Gd-ABC nanoparticles [58, 59]. Preparations for clinical trial are now underway beginning with cGMP manufacturing and preclinical toxicology testing. These Gd-ABC/ABCD nanoparticles are potentially excellent nanotechnology tools for the early detection and diagnosis of primary and metastatic cancer lesions. How effective remains to be seen when clinical trials can be performed. 

On the other hand, Müller et al. have described solid lipid nanoparticle (SLN) systems that represent genuinely alternative LNP systems [61–63]. Under optimised conditions, SLNs can carry MRI contrast agents [64], and SLNs containing [Gd-DTPA(H_2_O)]^2−^ and [Gd-DOTA(H_2_O)]^−^ have even been prepared for preclinical studies. 

Very recently, a multimodal imaging theranostic siRNA-ABC nanoparticle system (PEGylated siRNA-nanoparticle system) was described that had been assembled by the stepwise formulation of PEGylated cationic liposomes (prepared using Gd.DOTA.DSA and DOPE-Rhodamine amongst other lipids), followed by the entrapment of Alexa fluor 488-labelled antisurvivin siRNA. These nanoparticles were found able to mediate functional delivery of siRNA to tumours giving rise to a significant phenotypic (pharmacodynamic) reductions in tumour sizes relative to controls, while at the same time nanoparticle biodistribution (DOPE-Rhodamine fluorescence plus MRI) and siRNA pharmacokinetic behaviour (Alexa fluor 488 fluorescence) could be observed by means of simultaneous real-time imaging [45]. This concept of multimodal imaging theranostic nanoparticles for cancer imaging and therapy is certain to grow in importance in preclinical cancer nanotechnology studies and maybe too in the clinic.

## 4. Next Generation LNPs for Cancer Imaging and Therapy

Multimodal imaging theranostic nanoparticles may offer substantial benefits for cancer diagnosis and therapy going forward but only in combination with further advances in nanoparticle platform delivery technologies. What might these advances be and how might they be implemented? As far as imaging LNPs are concerned for detection of cancer, providing that all that is required for diagnosis is LNP accumulation within cancer lesions then current imaging nanoparticle technologies may well be sufficient. However, for personalized medicine to really take off, the detection of cancer disease specific biomarkers *in vivo* is really required. In order to achieve this, considerable attention may well have to be paid to the appropriate design and selection of ligands for the biological targeting layer (D-layer).

As far as LNPs for cancer therapy are concerned, the opportunities for delivery are relatively limited at this point in time, primarily due to the facile partition of current LNPs postadministration to liver and to solid tumours* in vivo* and in clinic. In order to enable partition to other organs of interest and even to diseased target cell populations within, there is now an imperative to introduce new design features involving new tool-kits of chemical components. Clearly the design of these new tool-kits of chemical components should be informed by rules for the control of nanoparticle biodistribution and API pharmacokinetics. Such rule sets are emerging but may take several years yet to become fully or even sufficiently understood. In addition, there are other issues. For instance, the central ABCD nanoparticle paradigm has a primary design weakness in that the stealth biocompatibility polymer layer (typically PEG-based) (C-layer) does not prevent nanoparticle entry into cells but may substantially inhibit functional intracellular delivery of the therapeutic agent, unless sufficiently removed by the time of target cell-entry or else during the process of cell-entry. Hence, overcoming the C-layer paradox should be a primary focus for ABCD nanoparticle development over the next few years. In this respect, there has been a growing interest in the concept of nanoparticles that possess the property of triggerability. Such nanoparticles are designed for high levels of stability in biological fluid from points of administration to target cells whereupon they become triggered for the controlled release of therapeutic agent payload(s) by changes in local endogenous conditions (such as in pH, *t*_1/2_, enzyme, redox state, and temperature status), [42–46, 65] or through application of an external/exogenous stimulus (Wright M. et al., 2013, papers in preparation and submission). While much of previous work on this topic has revolved around change(s) in local endogenous conditions [42–46, 65], the development of appropriate exogenous stimuli looks to be a real growth area for the future. In principle, all ABC/ABCD nanoparticles could be triggered to exhibit physical property change(s) through interaction with light, ultrasound, radiofrequency, and thermal radiation from defined sources. So how might this be harnessed?

Today, the journey to triggered, multimodal imaging theranostic drug nanoparticles for cancer therapy appears well underway. A few years ago, a thermally triggered drug-ABC nanoparticle system (thermally triggered PEGylated drug nanoparticle system, now known as ThermoDox, Celsion) was described based upon Doxil. ThermoDox nanoparticles were formulated using lipid compositions that included lyso-phospholipids in order to encapsulate doxorubicin within thermosensitive lipid bilayer membranes [66, 67]. At induced temperatures above 37°C, these membranes were observed to become porous allowing for substantial controlled local drug release. Needham et al. were first to demonstrate the use of such thermally triggered drug-ABC nanoparticles for the controlled local release of drug into target tissues* in vivo* [68], thus allowing for the treatment of tumours more efficiently than was achieved following administration of the thermally insensitive, Doxil parent system [69]. ThermoDox is currently the subject of phase III HEAT studies and phase II ABLATE studies. In the latter studies, ThermoDox was administered intravenously in combination with radio frequency ablation (RFA) of tumour tissue. In this case, the RFA acts as an exogenous source of local tissue hyperthermia (39.5–42°C) that simultaneously acts as a thermal trigger for controlled release of ThermoDox encapsulated doxorubicin. The company's pipeline going forward focuses on the use of Thermodox nanoparticles under thermal triggered release conditions for the treatment of breast, colorectal, and primary liver cancer lesions [70, 71]. This is the first time that thermally triggered drug-ABC nanoparticles have been devised and used in clinical trials.

**Figure 3:**
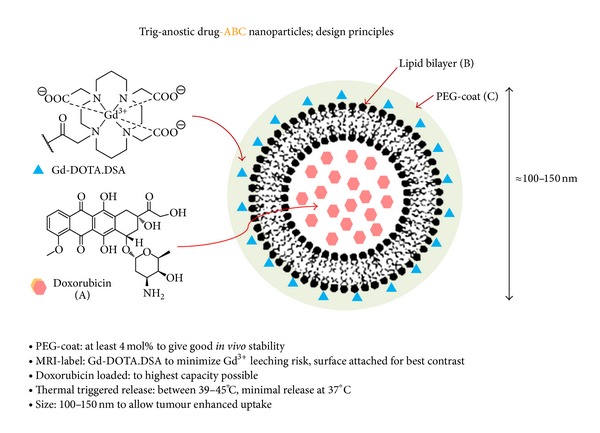
Schematic of thermal trig-anostic drug-ABC nanoparticles (thermal TNPs) enabled for thermally triggered release of encapsulated drug in tumours by means of ultrasound, together with real-time, diagnostic imaging of nanoparticle biodistribution by MRI with drug pharmacokinetics.

A further evolution of this concept has now been more recently reported with the simultaneous entrapment of both doxorubicin and an MRI positive contrast agent, Gd(HPDO_3_A)(H_2_O), into thermally triggered drug-ABC nanoparticles [72]. High frequency ultrasound (HIFU) was used as an alternative thermal trigger for the controlled release of encapsulated drug at 42°C. The simultaneous release of MRI contrast agent enabled the observation of release in real time and led to an estimation of doxorubicin release kinetics. Researchers involved in ThermoDox have similarly reported on the development of a thermally triggered drug-ABC nanoparticle system with doxorubicin co-encapsulated with the MRI contrast agent Prohance [73]. Using HIFU as a thermal trigger once more, they were able to promote controlled release of drug in rabbits with Vx2 tumours and monitor drug release in real time by MRI [74]. The same researchers also developed an algorithm to simulate the thermal trigger effects of HIFU [75]. Simulation data were in agreement with the HIFU-induced mean tissue temperature increasing from 37°C to between 40.4°C and 41.3°C, leading to quite heterogeneous kinetic drug release behaviour [75]. On the other hand, we have striven to draw inspiration from the Gd-ABC and Gd-ABCD imaging nanoparticle systems described above [58–60, 76, 77] and ThermoDox data, in order to derive alternative thermally triggered theranostic drug-ABC nanoparticles. These could also be described as thermal trig-anostic drug-ABC nanoparticles shortened to the acronym thermal TNPs (Figure 3). 

By description, these nanoparticles are enabled for thermally triggered release of encapsulated drug in tumours by means of ultrasound, together with real-time, diagnostic imaging of nanoparticle biodistribution with drug pharmacokinetics. Critical to this proposition is the use of Gd.DOTA.DSA once again. Going forward, MRI agent use could be supplemented with other substantive clinical imaging agents leading to new families of triggered multimodal imaging theranostic drug-ABC nanoparticles. An alternative description for such nanoparticles might be trig-anostic^*n*^ drug-ABC nanoparticles where *n* is number of clinical imaging modes employed, a description that could then be shortened to the acronym ^*n*^TNPs.

Following this, the ultimate would be the realization of targeted trig-anostic^*n*^ therapeutically multifunctional drug-ABCD nanoparticles. These might be described alternatively as targeted trig-anostic^*n*^  drug^*m*^-ABCD nanoparticles where *m* is the number of active therapeutic agents encapsulated/entrapped, a description that reduces to the simple acronym of targeted ^*n*^T_*m*_NPs. Indeed some nanoshell structures have recently been reported predoped with MRI probes (by introduction of a 10 nm iron oxide layer over the silica core) and/or NIR probes (indocyanine green dye), then set up (with streptavidin) for surface conjugation of anticancer antibodies (biotin labelled) plus the surface postcoupling (disulphide bond formation) of a PEG biocompatibility layer. The result could be described directly as a targeted trig-anostic^2^ drug^2^-ABCD nanoparticle system (i.e., targeted ^2^T_2_NP system) created with the capability for real time MRI and NIR contrast imaging in combination with the capacity for anti-HER-2 chemotherapy and photothermal ablation therapy (post illumination with 808 nm wavelength NIR laser) both *in vitro* and *in vivo* [78, 79]. The LNP equivalent is now awaited.

## 5. Conclusions and Future Perspective

Nanotechnology is revolutionising research and development in healthcare. Currently, the most advanced clinical grade nanotechnologies in cancer are LNPs. Unfortunately there remains scepticism from the big pharma industry and from clinicians themselves regarding the efficacy and safety of such nanoparticle technologies. Such scepticism will only be solved with the advent of reliable cGMP-grade manufacturing processes and reliable preclinical ADME/toxicology data, followed by a range of successful first-in-man studies. While these data are being acquired, nanoparticle technologies continue to be innovated in the laboratory. The ultimate push will be for targeted trig-anostic^*n*^  drug^*m*^-ABCD nanoparticles (targeted ^*n*^T_*m*_NPs) that are enabled for targeted delivery then triggered release of *m* active therapeutic agents (or drug entities), all monitored by simultaneous, real-time diagnostic imaging using *n* different imaging agent probes integrated into the nanoparticle. Of the latter, both NIR and ^19^F-NMR spectroscopy probes [80] could have real clinical potential alongside MRI. Such multiplicity of functions offers the very real opportunity for highly personalized drug nanoparticles assembly from selected tool-kits of chemical components, highly refined for specific, personalized delivery applications. As this vision begins to take shape, so we will be looking on a very different world of innovative, interactive healthcare products with vastly more potential to treat and even to cure cancer than has ever been seen before. 

Inevitably, words of balance and caution need to be expressed as well. This review has focused on LNPs and particularly on those that conform to the ABCD nanoparticle structural paradigm. There is plenty enough good reason for this focus given prospects for LNPs that conform to this paradigm *in vivo*, in pre-clinical studies and even in clinic. However, nanoparticles now come in many shapes and sizes ranging from polymer-based nanoparticles (PNPs) to hard, inorganic nanoparticle structures, such as the highly novel and advanced targeted ^2^T_2_NP system mentioned above. However, in general, although many such systems are showing promise *in vivo*, few PNPs or inorganic nanoparticle structures have advanced significantly towards clinical applications. My own view is that many of these technologies may induce significant toxicologies in humans, not seen with LNP systems; therefore, substantial preclinical evaluation would be essential and clinical trials would need to be performed with extreme caution in these cases. Accordingly, my expectation is that LNPs should be the first nanoparticle systems to make a substantial impact on cancer nanotechnology going forward and on the management of cancers in general. Therefore, Doxil nanoparticles should be seen as just the first of a wave of exciting new LNP-mediated drug delivery products that could have a truly transformational impact on anticancer therapeutics and diagnostics in the years to come.
